# Host learning selects for the coevolution of greater egg mimicry and narrower antiparasitic egg-rejection thresholds

**DOI:** 10.1093/evlett/qrad041

**Published:** 2023-09-21

**Authors:** Kuangyi Xu, Maria R Servedio, Sarah K Winnicki, Csaba Moskat, Jeffrey P Hoover, Abbigail M Turner, Mark E Hauber

**Affiliations:** Department of Biology, University of North Carolina, Chapel Hill, NC, United States; Department of Biology, University of North Carolina, Chapel Hill, NC, United States; Department of Evolution, Ecology, and Behavior, School of Integrative Biology, University of Illinois, Urbana–Champaign, Champaign, IL, United States; Program in Ecology, Evolution, and Conservation, University of Illinois, Urbana–Champaign, Champaign, IL, United States; Hungarian Natural History Museum, Budapest, Hungary; llinois Natural History Survey, Prairie Research Institute, University of Illinois, Urbana–Champaign, Champaign, IL, United States; Department of Evolution, Ecology, and Behavior, School of Integrative Biology, University of Illinois, Urbana–Champaign, Champaign, IL, United States; Department of Evolution, Ecology, and Behavior, School of Integrative Biology, University of Illinois, Urbana–Champaign, Champaign, IL, United States; Program in Ecology, Evolution, and Conservation, University of Illinois, Urbana–Champaign, Champaign, IL, United States; llinois Natural History Survey, Prairie Research Institute, University of Illinois, Urbana–Champaign, Champaign, IL, United States; Advanced Science Research Center and Program in Psychology, Graduate Center of the City University of New York, New York, NY, United States

**Keywords:** brood parasitism, experience dependence, learning, obligate, recognition threshold

## Abstract

Egg rejection is an effective and widespread antiparasitic defense to eliminate foreign eggs from the nests of hosts of brood parasitic birds. Several lines of observational and critical experimental evidence support a role for learning by hosts in the recognition of parasitic versus own eggs; specifically, individual hosts that have had prior or current experience with brood parasitism are more likely to reject foreign eggs. Here we confirm experimentally the role of prior experience in altering subsequent egg-rejection decisions in the American robin *Turdus migratorius*, a free-living host species of an obligate brood parasite, the brown-headed cowbird *Molothrus ater*. We then model the coevolutionary trajectory of both the extent of mimicry of host eggs by parasitic eggs and the host’s egg rejection thresholds in response to an increasing role of learning in egg recognition. Critically, with more learning, we see the evolution of both narrower (more discriminating) rejection thresholds in hosts and greater egg mimicry in parasites. Increasing host clutch size (number of eggs/nest) and increasing parasite load (parasitism rate) also have narrowing effects on the egg-rejection threshold. Together, these results suggest that learning from prior experience with egg rejection may play an important role in the coevolution of egg-mimetic lineages of brood parasites and the refined egg rejection defenses of hosts.

## Introduction

In avian obligate brood parasitism, parasites lay their eggs in the nests of other species and hosts care for the genetically unrelated propagules of parasites (Soler, 2014; Winfree, 1999). The host’s misdirected parental care imposes a fitness cost by reducing their brood’s survival and success (Hauber, 2003). Many host lineages have evolved foreign-egg rejection as an effective antiparasitic defense to reduce these costs, whereby they recognize and eliminate the parasitic egg (Davies, 2000). For example, hosts of the most mimetic Common Cuckoo *Cuculus canorus* host-races (or *gentes*) are best at rejecting foreign eggs from their nests (60–100% rejection rate), whereas hosts of less-mimetic eggs reject fewer foreign eggs (0–40%), leading to a positive coevolutionary pattern between host–parasite egg appearance mimicry and host egg rejection (Stoddard & Stevens, 2010, 2011). In contrast, hosts of some generally less-mimetic parasitic species are even poorer at rejecting foreign eggs (e.g., <30%, as seen in non-rejecter hosts of the non-mimetic Brown-headed Cowbird *Molothrus ater*; Rutledge et al., 2021). Nonrejection and tolerance of imperfectly (Dixit et al., 2023) or fully non-mimetic (Rutledge et al., 2021) parasitic eggs (Medina & Langmore, 2016) may be due to the relative historical recency of the onset host–parasite interactions in novel, human-modified habitats and ongoing species-range expansions (i.e., evolutionary lag hypothesis) or it may be an outcome of balancing selection between rejection costs (mistakes and mishaps) and the fitness benefits of successful rejections of parasitic propagules (the evolutionary equilibrium hypothesis) (Soler, 2014, Turner et al., 2021; Winfree, 1999).

There has been extensive previous modeling (Lotem, 1993; Rodriguez-Girones & Lotem, 1999; Stokke et al., 2007; Svennungsen & Holen, 2010) to address the role of experiential learning (sensu Barron et al., 2015) about the birds’ own eggs in their clutch(es) in the process of recognizing and rejecting foreign eggs. Most of these modeling approaches to learning about parasitic egg phenotypes rely on signal detection theory (e.g., Davies et al., 1996) and its implications for conspecific recognition through optimal acceptance theory (Reeve, 1989; Scharf et al., 2020). In turn, empirical work (Davies et al., 1996; Lotem et al., 1992; Molina-Morales et al., 2014; Moskat et al., 2014; Moskat & Hauber, 2007; Strausberger & Rothstein, 2009, but see Noh et al., 2018) demonstrated that through experience-dependent mechanisms hosts can gain reliable information about the acceptable suite of (self) egg phenotypes in their current and subsequent clutches (Spottiswoode & Busch, 2019 but see Hauber et al., 2020). Alternatively, or in addition, hosts may be able to learn about the identity and phenotype of parasitic adults (Feeney & Langmore, 2013; Thorogood & Davies, 2013), or even parasitic eggs (Yang & Feeney, 2022), from conspecifics through socially acquired information. Critically, the results seen in empirical studies are also predicted by signal detection theory itself (e.g., Hauber et al., 2006; reviewed by Ruiz-Raya & Soler, 2020).

Specifically, however, experimental support for the experiential egg-learning by first-time breeding, naive hosts is sparse, but what little is available shows some role of experience-dependence; specifically when hosts face a parasite-like egg as the first egg in their clutch, they subsequently accept more foreign eggs (Moskat & Hauber, 2007; Strausberger & Rothstein, 2009, but see Amundsen et al., 2003). This is consistent with the hypothesis that naïve breeders learn about their own eggs during their first, rather than during each subsequent, breeding attempt (Moskat et al., 2014). Nonetheless, multiple and repeated parasitism within the same and across different clutches, respectively, is common across diverse host–parasite systems, including cuckoos (Manna et al., 2019) and cowbirds (Hoover, 2003). To face these repeated parasitism contexts, some hosts of brood parasites have also evolved learning mechanisms whereby they inspect and acquire recognition templates from each clutch de novo (e.g., Soler et al., 2013) through template updating mechanisms that rely on repeated learning (reviewed by Ruiz-Raya & Soler, 2020).

More recently, conceptual reviews of egg rejection have taken into consideration the potential roles of individual learning and experience-dependent plasticity in shaping the course and outcome of host egg mimicry by brood parasites and the evolution of foreign-egg rejection thresholds in hosts (e.g., Ruiz-Raya & Soler, 2020; Spottiswoode & Busch, 2019) but detailed modeling of the role of such learning is still lagging. Initial intuition might predict that when recognition can be improved by learning, the genetic evolution of improved discrimination might be inhibited (e.g., as occurs for conspecific mate recognition in Servedio & Dukas, 2013, also Price et al., 2003, but see Sznajder et al., 2012). Nonetheless, in his species-recognition model, Reeve, (1989) predicted that repeated exposure to (and, hence, experience with) heterospecifics should in turn cause a narrowing of the coevolved acceptance thresholds (i.e., more egg rejection). Here, we first explicitly test for experimental evidence of experience-dependent narrowing shifts in rejection behavior by a free-living host of an obligate brood parasite. We then model the evolutionary role of this learning, specifically asking how it shapes the outcomes of the coevolutionary arms-race between hosts and parasites.

## Methods

### Experimental test

To test the assumption that prior experience influences subsequent egg rejection behavior in hosts of avian brood parasites, we studied a free-living population of American robins, *Turdus migratorius*, in Champaign County, IL, USA. Birds at our site breed in open cup nests built in ornamentals on tree farms and other private properties. Robins are rejecters of natural or model cowbird eggs (>90%: Luro et al., 2018; Rothstein, 1982) but within 1 day they reject deep blue (indigo) model eggs only at intermediate rates (e.g., 42%: Abolins-Abols & Hauber, 2020a; 54%: Hauber et al., 2020). Here we relied on this intermediate rejection rate of this deep blue egg type to be able to predictably and measurably increase the robins’ rejection responses to it following prior experimental experience with cowbird-like eggs.

We monitored active robin nests to confirm clutch completion (two subsequent days of nest visits with the same clutch size) and then exposed each nest to one of two treatments with model eggs. This meant that the nests were parasitized both in the first and second instance during the incubation stage, instead of the laying stage when brood parasitism typically occurs (e.g., Geltsch et al., 2016). However, in robins, the nesting stage does not covary with egg rejection propensity (Abolins-Abols & Hauber, 2020b; Croston & Hauber, 2014).

Model eggs were manufactured as per Igic et al. (2015) and sourced commercially from Shapeways.com as the “Cow Bird” egg model. In turn, the paint mixes followed the recipes for robin blue, quail-beige, and quail-spotted brown colors from Canniff et al. (2018). For deep blue eggs, we used Winsor and Newton Galeria Ultramarine Blue acrylic paint; all paint was applied in three coats.

The first treatment involved nests that received a 3D-printed cowbird egg-sized, -shaped, and -weighted model egg painted a light blue color designed to mimic the robin’s own egg color. Nests in the second treatment group received the same type of 3D-printed model egg but painted quail-beige and speckled with a quail-speckle brown color to resemble a natural cowbird egg. We assessed whether model eggs were rejected 1 day after their deployment, removed any non-ejected eggs, and immediately placed a deep blue model egg in each nest. The rejection of a deep blue egg in the same nest was again assessed 1 day later. Nest abandonment is not a response by robins to experimental parasitism (Croston & Hauber, 2014), therefore both depredated and abandoned nests were removed from the analyses.

Treatments were assigned randomly, and so we primarily analyzed the egg rejection data using Fisher’s exact tests. This was justified as nests between two treatments did not vary in their experimental dates (*t* = −0.25, *p* = .80) or their clutch sizes (*t* = 0.10, *p* = .92). Nonetheless, we also ran a nominal logistic analysis with both date and clutch size included as predictors alongside the treatment, and egg rejection outcome as the bivariate response variable. We initially included all the pairwise interaction terms of the predictors in the model but none of these were significant (all *p* > .7). For future meta-analytical purposes, we also calculated the odds ratio of each experimental outcome. For both the Fisher’s exact test and the nominal logistic analysis we predicted higher egg rejection rates of the deep blue eggs following the prior exposure to the cowbird-like treatment eggs versus the robin-mimetic blue treatment eggs.

### Modeling

We consider a single bird species that is the sole host of a population of cowbirds. For mathematical simplicity and tractability, we assume that host females lay two clutches during their lifetime and examine the evolutionary effects of altering rejection rates in the second clutch due to learning, in differing amounts, from an experience of egg rejection during the first clutch. Specifically, we assume that hosts that encounter an egg unusual enough to reject in their first clutch undergo sensitization (a form of non-associative learning, Shettleworth, 2010), which lowers their threshold for egg rejection during a repeat of the stimulus (of an unusually large egg) if one appears in the second clutch. Note that our empirical study focused on experience-dependence within the same brood (e.g., Samas et al., 2011), although experiments also have been conducted on the repeatability of egg rejection across broods in other parasite–host species (e.g., Grim et al., 2014; Molina-Morales et al., 2014).

**Figure 1.**
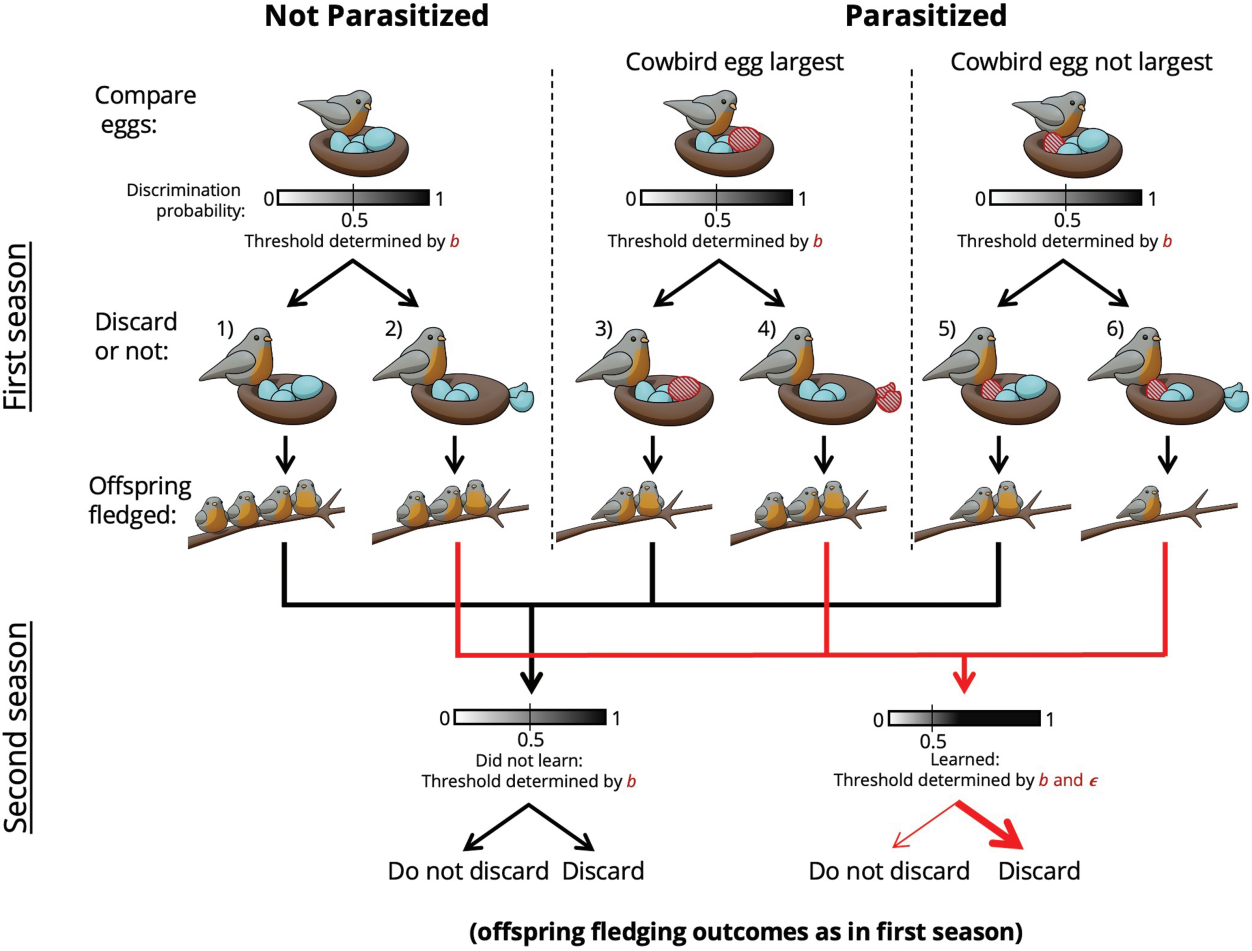
Schematic of the mathematical model. Cowbird eggs in this figure have red diagonal lines to identify them for the reader, but to the birds they are visually identical to host eggs, other than in their size. Irrespective of whether there is parasitism or not, the host compares the size of the largest and second largest egg in the nest. The probability that the host will discard the largest egg increases as the difference in size between these two eggs becomes larger. This probability is marked “Discrimination probability” and is shown by the gray scale bar in the step called “compare eggs.” How sensitive the host is to the phenotypic difference is measured by the discrimination threshold *b*, which corresponds to the critical phenotypic difference when the probability of discarding the largest egg is 50%, shown by a thin vertical bar. Depending on whether or not parasitization occurred and the relative sizes of the eggs in the nest, one of six possible outcomes ensue (seen from left to right on the figure). (1) If the host is not parasitized and does not discard an egg, chicks will fledge from all *n* eggs in the nest (in this figure, *n* = 4). (2) If the host is not parasitized and discards an egg, *n* – 1 chicks will fledge. If the host is parasitized, one of the host eggs is replaced by the cowbird egg, and the cowbird egg may either be the largest in the nest or one of the remaining host eggs may be larger than the cowbird egg. (3) If the cowbird egg is largest and the host does not discard the largest egg, the host chicks will fledge with survival probability *s* (shown on this figure as ~0.66). (4) If the cowbird egg is largest and the host discards the largest egg, again *n* – 1 chicks will fledge. (5) If the cowbird egg is not the largest and the host does not discard an egg, the host chicks will again fledge with survival probability *s*. (6) Finally, if at least one of the *n* – 1 host eggs is larger than the cowbird egg and the host discards the largest egg, each of the remaining *n* – 2 host chicks will fledge with survival probability s. Learning occurs as follows. If a host does not discard an egg (first, third, and fifth columns, black pathways), the discrimination threshold of the host does not change and thus is still *b* in the second season (the events in the second season would thus look identical to those in the first season). In contrast, if the host does discard an egg (second, fourth, and sixth columns, red pathways), its discrimination threshold in the second season is reduced by an amount ϵ to b − ϵ. In this case, hosts will be more sensitive to the phenotypic difference between the eggs and would thus discard eggs more often (shown by the wider red arrow pointing to “discard” on the far right of the second season). The number of offspring fledged from the nests with discarded and not-discarded eggs would again proceed as in the first season. Artwork by Adrian Willett.

Our model roughly follows that of Servedio and Lande (2003). During a nesting season, each host female lays a single clutch of n eggs (see Table 1 for the biological meaning of key symbols). We varied the host clutch size (n) in our model as it has been known to impact egg rejection rates both comparatively and intraspecifically through Weber’s law (Akre & Johnsen, 2014; Dixit et al., 2021; Hauber et al., 2022). Following Servedio and Lande, (2003), and for simplicity of exposition, we use the term “‘size’” as a proxy for the appearance of the egg (size is also an important cue used by hosts for egg recognition, Samas et al., 2021). This can be interpreted literally as size but could also represent any avian-perceivable appearance of the egg as related to an egg-rejection decision, including size, shape, background color (light wavelength), and spotting pattern. Since egg size (and the majority of other traits for which it is a proxy) is effectively controlled by multiple loci, we treat it as a continuous trait. Specifically, the host egg size, $z_{h}$, has a normal distribution within a nest $f_{h}$($z_{h}$), with mean ${\bar{z}}_{w}$ and variance $\frac{n-1}{n}\sigma_{w}^{2}$. The within-nest mean ${\bar{z}}_{w}$ has a normal distribution $f_{w}\left({\bar{z}}_{w}\right)$, among nests, with mean ${\bar{z}}_{h}$ and variance $\sigma_{B}^{2}+\frac{\sigma_{\omega }^{2}}{n}$, where $\sigma_{B}^{2}$ and $\sigma_{w}^{2}$ are the between- and within-family variance in infinitely large families. When host nests are parasitized by cowbirds, we assume only one cowbird egg per nest (which is typical in many populations of parasitized cowbird hosts; e.g., Hauber et al., 2012; McLaren et al., 2003 but see Hoover, 2003). The number of eggs laid by each cowbird is $n_{c}$, and the size of cowbird eggs, $z_{c}$, also has a normal distribution $f_{c}\left(z_{c}\right)$, with mean ${\bar{z}}_{c}$ and variance $\sigma_{c}^{2}$. The model fully accounts for all possibilities of hosts being parasitized or not, having the cowbird egg constitute the largest egg in the nest or not, and a host discarding an egg or not, as shown in Figure 1. We present key assumptions leading to the derivation of selection differentials on host egg size $z_{h}$, cowbird egg size $z_{c}$, and a discrimination threshold of the smallest difference between egg sizes that will tend to trigger the host to discard an egg, b. Full derivations of the discrete-time recursion equations can be found in Supplementary Materials.

**Table 1.** Summary of biological meanings of key symbols used in the model.

Key parameters	Biological meaning
n	Number of host eggs in a single clutch
$f_{h}(z_{h})\;,\;{\bar{z}}_{w},\;\frac{(n-1)\;\sigma_{w}^{2}}{n}$	Distribution of host egg size, its mean, and variance within nests
$f_{w}({\bar{z}}_{w})\;$ , ${\bar{z}}_{h}$, $\sigma_{B}^{2}$+$\frac{\sigma_{w}^{2}}{n}$	Distribution of ${\bar{z}}_{W}$ among nests, its mean, and variance
$f_{c}(z_{c})\;$ , ${\bar{z}}_{c}$, $\sigma_{c}^{2}$	Distribution of cowbird egg size, its mean, and variance
C	Number of cowbird females
$n_{c}$	Number of eggs laid by each cowbird
H	Number of host females
$\nu^{2}$	Strength of discrimination
b	Discrimination threshold
$f_{b}(b)$ , $\bar{b}$, $\sigma_{b}^{2}$	Distribution of the threshold difference, its mean, and variance
s	Survival probability of a host egg if the nest is successfully parasitized
ϵ	Sensitivity of discrimination threshold shift in response to the egg size difference between the largest and second largest eggs
$\delta (z_{1},z_{2})\;$	Probability of discarding the largest egg, given that the sizes of the largest and the second largest eggs are $z_{1}$ and $z_{2}$
$\phi (z_{r},n)\;$	Distribution of the largest value from a distribution (extreme value distribution)
$D(z_{1},z_{2})\;$	Shift of threshold given the largest and the second largest eggs are $z_{1}$ and $z_{2}$
$z_{h},z_{r},z_{c}$	Size of the focal egg, the largest egg among the rest of the host eggs, the cowbird egg

We assume physiological stabilizing selection on egg size for both hosts and cowbirds. For hosts, the viability of an egg changes with the egg size $z_{h}$ as $w_{h}^{\;p}\left(z_{h}\right)=\exp\left[-\frac{{\left(z_{h}-\theta_{h}\right)}^{2}}{2{\omega_{h}}^{2}}\right]$, where $\theta_{h}$ is the fitness optimum, and $1/\omega_{h}^{2}$ measures the strength of viability selection. Similarly, the viability of a cowbird egg is $w_{c}^{\;p}\left(z_{c}\right)=\exp\left[-\frac{{\left(z_{c}-\theta_{c}\right)}^{2}}{2{\omega_{c}}^{2}}\right]$.

We denote the number of host and cowbird females by *H* and *C*, respectively. Cowbirds parasitize nests randomly, so that the probability that a nest is parasitized is $\frac{Cn_{c}}{H}$. This is a proxy for both per capita parasitism pressure in the population, which varies widely both within and between species, and the likelihood of encountering a cowbird laying in the nest which is known to impact antiparasitic behaviors in some cowbird and other hosts (e.g., Strausberger & Hauber, 2017; also Davies et al., 1996, but see Soler et al., 2000).

Before a cowbird lays an egg in a nest, it removes a random host egg (Sealy, 1992), reducing the number of host eggs in the nest to n−1 (Figure 1). After the population of breeding- (and parasitism-) naive individuals is parasitized, each host female compares the size of the largest ($z_{1}$) and second largest ($z_{2}$) eggs in her nest and discards the largest egg with probability (Stevens, 1975, pp. 182–186, Servedio & Lande, 2003)


$$\begin{array}{l} \begin{array}{l} \delta \left(z_{1},z_{2}\right)=\frac{1}{2}erf\left(\frac{z_{1}-z_{2}-b}{\sqrt{2}\nu }\right)\;\;,\left(z_{1}>z_{2}\right)\;,\; \end{array} \end{array}$$ (1)

where erf(⋅) is the error function (see gradient bars in Figure 1).

We expect our qualitative results to generalize to comparisons of the largest egg with other aspects of the clutch (e.g., the mean egg size). In Equation (1), the probability of discarding an egg increases quickly with the size difference $z_{1}-z_{2}$ when this size difference is close to a threshold value b (Davies et al., 1996). Specifically, when $z_{1}-z_{2}=b$, $\delta \left(z_{1},z_{2}\right)=\frac{1}{2}$. The parameter ν regulates how quickly the probability of discarding an egg increases with the size difference. We assume that the threshold value b has a normal distribution $f_{b}\left(b\right)$ among females, with mean $\bar{b}$ and variance $\sigma_{b}^{2}$. A nest is successfully parasitized if the cowbird egg is not discarded, and compared with a not-parasitized nest, the survival probability of a host egg in a successfully parasitized nest is s (shown as fewer fledglings from nests with a remaining cowbird egg in Figure 1).

We assume that each host female learns to lower its threshold value b once it considers that it has recognized a cowbird egg and hence has discarded an egg (regardless of whether it has discarded a cowbird egg or one of its own eggs). The decrease of the threshold value, D, has a linear dependence on the size difference between the largest and the second largest eggs, given by


$$\begin{array}{l} \begin{array}{l} D\left(z_{1},z_{2}\right)=-\epsilon \left(z_{1}-z_{2}\right)\;,\;\left(z_{1}\geq z_{2}\right)\;,\; \end{array} \end{array}$$ (2)

where ϵ measures the sensitivity of the discrimination threshold shift in response to the egg size difference (see gradient bar in bottom right of Figure 1). We assume there is no variation of ϵ in the population. Since the discrimination threshold due to learning from the first clutch cannot be transmitted to the offspring generation, we must track the joint distribution of the mean egg size of the female ${\bar{z}}_{w}$, the original discrimination threshold b, and the threshold after learning $b^{\prime }$, $P\left({\bar{z}}_{w},b,b^{\prime }\right)$.

To calculate the selection differential on a trait, the derivation of which is presented in full in the Supplementary Materials for host egg size $z_{h}$, cowbird egg size $z_{c}$, and the discrimination threshold b, we must obtain an expression describing how the mean fitness depends on the trait value. For example, for selection on the host egg size $z_{h}$, denoting the mean fitness of eggs with size $z_{h}$ during the first and second clutch by $w_{h}^{\left(1\right)}\left(z_{h}\right)$ and $w_{h}^{\left(2\right)}\left(z_{h}\right)$, respectively, the selection differential is


$$\begin{array}{l} \begin{array}{l} S_{h}=\frac{\overset{\infty }{\underset{-\infty }{\int }}z_{h}\left(w_{h}^{\left(1\right)}\left(z_{h}\right)+w_{h}^{\left(2\right)}\left(z_{h}\right)\right)f_{h}\left(z_{h}\right)dz_{h}}{\overset{\infty }{\underset{-\infty }{\int }}\left(w_{h}^{\left(1\right)}\left(z_{h}\right)+w_{h}^{\left(2\right)}\left(z_{h}\right)\right)f_{h}\left(z_{h}\right)dz_{h}}-{\bar{z}}_{h}.\; \end{array} \end{array}$$ (3)

The model involves many multidimensional numerical integrations, causing the numerical simulation of the recursions runs to be exceedingly slow. To approximate the equilibrium value at which the variables will stop changing with time, we run the system for at least 1,000 generations and fit the data of the last 5 generations with the function


$$\begin{array}{l} \begin{array}{l} y=A_{0}+A_{1}e^{-\lambda t},\; \end{array} \end{array}$$ (4)

where $A_{0}$ is the approximated equilibrium value. We use equation (4) as the fitting function because at the neighborhood of the equilibrium, the dynamics of the system will be a linear combination of exponential growth (e.g., ${\bar{z}}_{h}\left(t\right)=A_{0}+A_{1}e^{-\lambda_{1}t}+A_{2}e^{-\lambda_{2}t}+A_{3}e^{-\lambda_{3}t}$), where $\lambda_{1},\lambda_{2},\lambda_{3}$ are eigenvalues of the Jacobian matrix for the three recursion equations of the mean trait values. As the system converges towards the equilibrium, the dynamics will be dominated by the smallest eigenvalue $\lambda_{1}$. This approximation is validated by comparing the fitted equilibrium value with the exact equilibrium value for some parameters.

## Results

### Experimental tests

**Figure 2.**
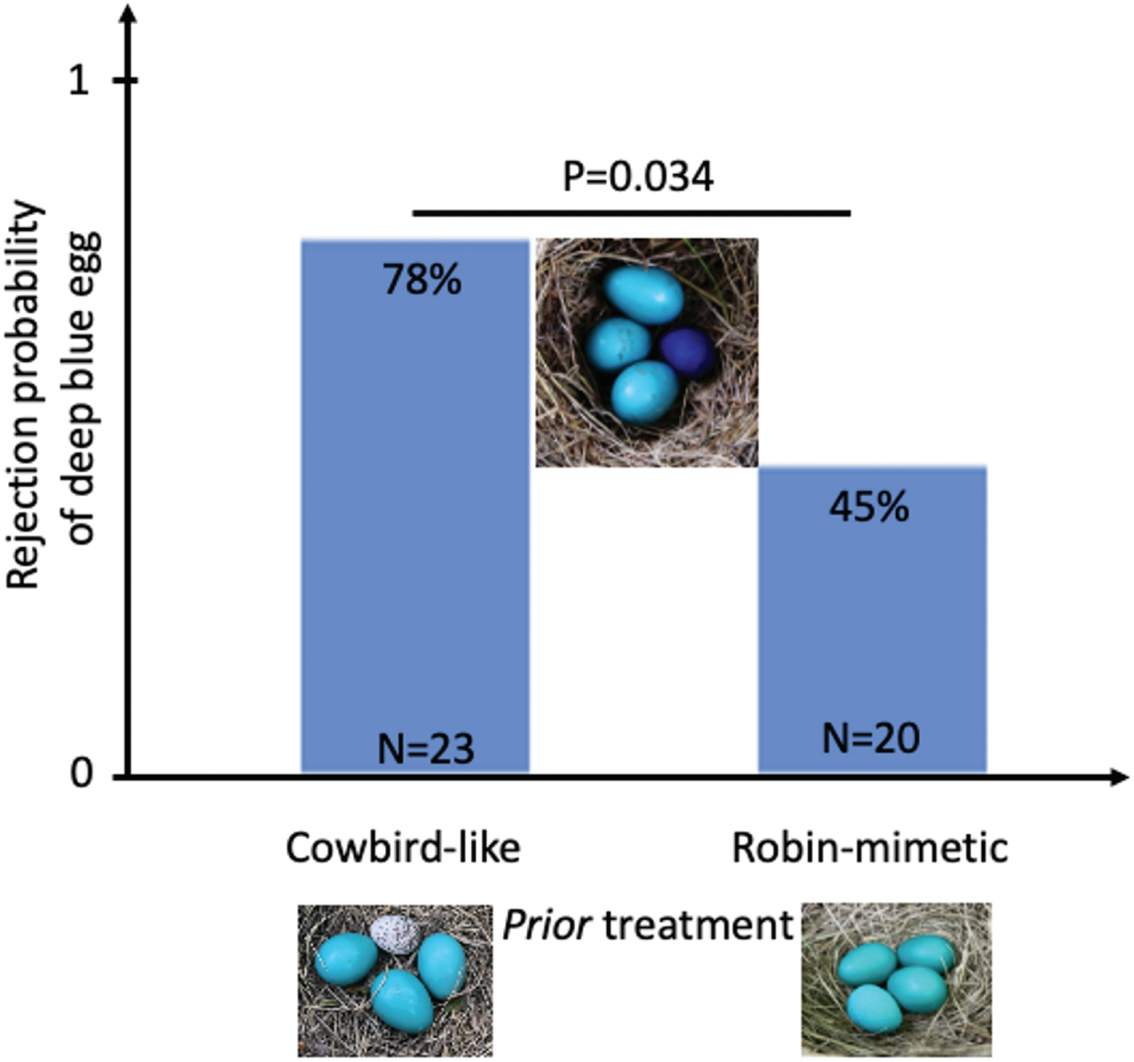
The proportions and percentages of the deep blue model eggs rejected from nests of American robins within 1 day following prior experience with a cowbird-like versus robin-mimetic model egg; the *p*-value indicates a significant treatment effect using a Fisher’s exact test.

As expected, cowbird-like treatment eggs were rejected (87.5%; 21 of 24 eggs) from the vast majority of robin nests, and also more so relative to robin-like eggs (9.5%; 2 of 21; *p* << .0001; odds ratio = 9.19). In addition, as predicted, deep blue model eggs were rejected more often following cowbird-like treatment eggs (18 of 23) versus robin-like treatment eggs (9 of 20; *p* = .0316; odds ratio = 1.74) (Figure 2). The nominal logistic model also revealed a significant effect of treatment only (χ^2^ = 6.01, *p* = .014) but not of date (χ^2^ = 2.64, *p* = .104) or clutch size (χ^2^ = 2.09, *p* = .148) overall.

### Modeling

**Figure 3.**
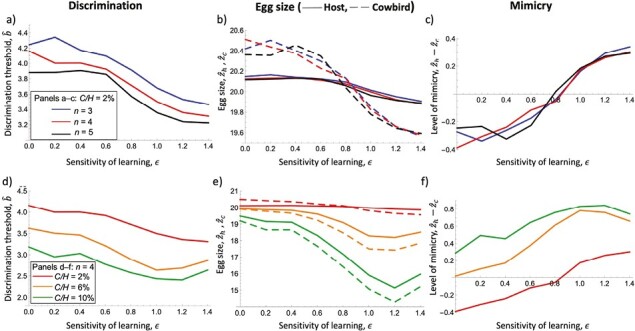
Effects of the sensitivity of learning on the equilibrium value of the discrimination threshold, egg size, and mimicry. (a–c) The effects of host clutch size. (d–f) The effect of parasitism rate (C/H). The left column shows the equilibrium value of the discrimination threshold $\hat{b}$, the middle column shows the equilibrium values of the host (solid) and cowbird (dashed) egg size, and the right column shows the amount of mimicry measured as the mean egg size difference between hosts and cowbirds $\left({\hat{z}}_{h}-{\hat{z}}_{c}\right)$. Other parameters used are as follows: $H=10^{5},\;n_{c}=10,\;s=0.7835,\;\sigma_{h}=\sigma_{b}=\nu =0.87,\;\sigma_{c}=1.3,\;\theta_{h}=20.235,\;\theta_{c}=24,\frac{\omega_{h}^{2}}{\sigma_{h}^{2}}=\frac{\omega_{c}^{2}}{\sigma_{c}^{2}}=100$.

We found a general lowering of the egg-rejection threshold (i.e., more discrimination and hence greater recognition of the parasitic egg in the clutch) with increased learning in our modeled coevolving system of hosts and parasites. The rejection threshold was also lowered with increased host clutch size (order of lines in Figure 3A) and increased parasite load (parasitism rate, as measured by cowbird to host population sizes, *C*/*H*, order of the lines in Figure 3D).

Additionally, as the amount of learned discrimination (ϵ)  increased from very low values, smaller cowbird eggs and host eggs evolved alongside the egg-rejection threshold in a coevolutionary arms race (Figure 3B and E). Critically, we found that cowbird egg size decreased more rapidly than host egg size, especially with low parasitism rates. As learning increases from very low values, this causes the extent of mimicry between parasite and host eggs to increase ($|{\bar{z}}_{h}-{\bar{z}}_{c}|$ becomes closer to zero), provided that the parasite load (*C*/*H*) is not too high (*C*/*H* = 2%, Figure 3C and F). However, when the extent of learned change (ϵ)  continues to increase to high levels, the rapidly evolving cowbird eggs “overshoot” the host egg size by becoming even smaller than the host egg (where the dashed and solid lines cross, Figure 3B). Because perfect mimicry is defined as having the egg sizes be identical, this overshooting causes mimicry $(|{\bar{z}}_{h}-{\bar{z}}_{c}|)\;$ to decrease as the egg sizes become more dissimilar again with very high learning. We also found decreasing mimicry when the parasite load (i.e., parasitism rate) was high (*C*/*H* = 6%, 10%). In this case, the cowbird egg size evolved to be smaller than the host egg size regardless of the amount of learned change in discrimination. These outcomes are likely unrealistic if hosts’ egg-rejection decision mainly relies on egg size in particular, as cowbird eggs in nature are generally larger than host eggs. They may be realistic for other aspects of avian-perceivable egg appearance (including shape, color, maculation, texture: Samas et al., 2021; Stoddard & Hauber, 2017; Turner et al., 2022) although as described in Discussion we expect the discrimination rule may evolve in this case.

As the cowbird eggs continued to decrease in size, the discrimination threshold *b* evolves to be even lower, in order to allow the host to discard cowbird eggs. However, when the learned shifts are too large (very high ϵ), a low discrimination threshold may lower individual fitness, since the cowbird egg size evolves to be, on average, smaller than the host egg size (Figure 3E), and thus changes by learning will increase the likelihood of a host discarding its own eggs. In this case, we can see evolved changes of the discrimination threshold start to reverse direction, causing less rejection of eggs in general (Figure 3D).

## Discussion

A clear empirical pattern of avian brood parasite–host coevolutionary arms-race is that hosts of the better egg mimetic brood parasite lineages are also superior in rejecting foreign eggs from the nests, whereas hosts of less-mimetic parasites are poorer at it (Davies, 2000). Accordingly, increasing avian-perceptual mimicry by brood parasites is positively related to the experimental rejection of model egg colors from the nests by the respective host species (Stoddard & Stevens, 2010, 2011). Such behavioral defenses are also paralleled by the evolution of greater sensitivity of the avian visual system (e.g., greater relative eye sizes) in some of these more-rejecting host species (Ausprey & Hauber, 2021). However, it has remained empirically unclear whether learning is involved in the antiparasitic defenses of host species since we are faced with mixed evidence for a role of learning in the experimental literature for the ontogenetic shifts in hosts’ egg rejection behaviors (e.g., no: Amundsen et al., 2003; yes: Lotem et al., 1995; Molina-Morales et al., 2014; Moskat et al., 2014). Furthermore, despite extensive modeling approaches (see Introduction), it has remained theoretically unclear to what general extent any learning or plasticity in egg rejection behavior may affect the evolutionary paths and outcomes of parasitic mimicry, host egg rejection, and their interaction.

Our empirical data provide strong supportive evidence that learning from prior experience of rejecting unusual eggs can promote egg rejection when foreign eggs are subsequently encountered. Specifically, here we demonstrate a strong experience-dependent component of egg rejection behaviors in American robins: when first exposed to cowbird-like eggs versus robin-like eggs, they are nearly twice as likely to reject the focal type (i.e., deep blue) of foreign model eggs on the subsequent day (Figure 2). This result is consistent with the predictions of signal detection theory (Reeve, 1989), which, when applied to brood parasitism, predicts a narrower (more discriminatory) egg acceptance threshold following prior exposure to the heterospecific phenotype (Davies et al., 1996; Hauber et al., 2006).

We further demonstrate, using analyses of the equilibrium of the coevolutionary process, that empirical patterns of more egg rejection by hosts of better mimics result from coevolutionary processes in a more pronounced fashion when incorporating learning into the models as a mechanism to refine egg-rejection thresholds at the level of individually impacted hosts. Specifically, moderate levels of changes in rejection through learning resulted in lower (more discriminatory) evolved thresholds, irrespective of host clutch sizes and parasite loads. Coevolution with the cowbird parasites also resulted in greater egg mimicry, provided that parasitism rates were not too high. Exceptions to these patterns were seen at high levels of learning and high parasitism rates, where egg phenotypes are such that self versus other discrimination breaks down and the hosts likely engage in recognition errors to mistakenly reject their own eggs disproportionately more often. Such recognition errors have been detected in empirical systems, including both in the contexts of ontogenetic experience dependence (by replacing naive hosts’ first-laid clutches with all model eggs and they end up rejecting their own eggs: Strausberger & Rothstein, 2009).

Regarding the role of greater host clutch sizes yielding narrower rejection thresholds and, at times, more mimicry, regardless of learning (the order of the lines in Figure 3, top), this theoretical prediction has mixed empirical support. At the level of individual hosts, Weber’s law predicts that smaller, rather than larger, clutch sizes should yield more discriminating host responses (Akre & Johnsen, 2014; Dixit et al., 2021). There is empirical evidence for this pattern both intraspecifically (e.g., Abolins-Abols & Hauber, 2020b) and comparatively (Hauber et al., 2022), but these single time-point patterns cannot be equated with the course of coevolutionary interactions as the cowbird eggs also evolve across time. In turn, regarding the role of a greater parasitism rate in increasing the accuracy of parasite–egg detection through lowering hosts’ rejection thresholds, the modeled pattern is intuitive in the sense that more selection by greater parasitism rate should result in more discriminating hosts (order of the lines, Figure 3, bottom).

The results of the model show that cowbird egg sizes can “overshoot” host egg sizes in their rapid decrease, meaning that the cowbird eggs become smaller than the host eggs. This finding may be unrealistic if hosts’ egg-rejection decision mainly relies on egg size in particular, but can still hold for a more general set of traits (remember that “size” is used as a proxy for egg appearance to discuss the model, but the modeling results apply to any phenotype). For example, the hue of a cowbird egg may change from being greater than that of a host egg to briefly mimicking that of the host egg for a short period of evolutionary time, but then may continue to evolve to be less than that of a host egg. However, in our model (where hosts reject the larger of the two largest eggs), the evolutionary outcome of “overshooting” occurs because it is extremely detrimental to the cowbird to have an egg that is unusually large in particular, as opposed to being unusual in size in general. Continuing to use size as an example, we expect that the evolutionary outcome of “overshooting” would induce selection pressure for hosts to change their criteria for acceptance of an egg such that they would discard a usually small egg instead of an unusually large one. Depending on the evolutionary flexibility in such criteria, we would expect anything from the system evolving worsening mimicry (if evolution of the criterion is constrained), through fluctuating between increasing and decreasing levels of mimicry (if evolution of the criterion occurs to switch between rejecting larger and smaller eggs but is slower than the evolution of egg size and discrimination), to remaining near a minimum level of mimicry (if the criterion can evolve rapidly or if there is a general criterion of discarding any unusual egg).

Overall, we found that variation in the extent of the role of learning about the host’s own eggs may modulate the outcome of coevolutionary interactions with respect to both shifting (lowering) egg-rejection thresholds and altering (increasing, for more modest levels of learning) the extent of host–parasite mimicry. Accordingly, these patterns have now been repeatedly detected along different axes of host–parasite mimicry empirically. However, the results predict that the recognition of foreign eggs will depend more extensively on first experience with their own eggs in hosts of the more mimetic brood parasites, a prediction that remains to be assessed in most host species of avian brood parasitic lineages.

## Supplementary Material

qrad041_suppl_Supplementary_Material

## Data Availability

The bivariate egg rejection data (yes/no) resulting from the randomized experiments are stated and illustrated in the manuscript. The mathematical details and derivations of the model are detailed in Supplementary Materials.
