# Supplementary material for: Host learning selects for the coevolution of greater egg mimicry and narrower antiparasitic egg-rejection thresholds
Source: Evol Lett. 2023 Sep 21;7(6):413–21. doi: 10.1093/evlett/qrad041 (PMC10693006; doi:10.1093/evlett/qrad041)
Supplement: qrad041_suppl_Supplementary_Material [file qrad041_suppl_supplementary_material.docx]

**Supplementary Materials for “Host learning selects for coevolution of greater egg-mimicry and narrower anti-parasitic egg-rejection thresholds”** – Kuangyi Xu, Maria R. Servedio, Sarah K. Winnicki, Csaba Moskat, Jeffrey P. Hoover, Abbigail M. Turner, and Mark E. Hauber

In the main text, we briefly describe the model setup, and here we present the detailed derivation of the model. The key point is to obtain the selection differentials for the three traits: host egg size, host discrimination threshold, cowbird egg size. The equilibrium state is when three selection differentials equal to 0.

Considering a randomly chosen host egg $i$ from a randomly chosen nest, given that the size of egg $i$, $x_{i}$, is $z_{h}$, the probability that egg $i$ survives is

$$\begin{aligned} w_{h}^{d}\left( z_{h}|\overline{z}_{w},b \right)= Pr \left[ \mathrm{egg}i \mathrm{survives} | x_{i}=z_{h} \right]=\left( 1-\frac{Cn_{c}}{H} \right)\Pr\left[ \mathrm{egg}i\mathrm{survives} \right|x_{i}=z_{h}, not parasitized]+ \\ \frac{Cn_{c}}{H}\left( 1-\frac{1}{n} \right)\Pr\left[ \mathrm{egg}i\mathrm{survives} \right|x_{i}=z_{h},parasitized],\#\left( S1 \right) \end{aligned}$$

where the definitions of the parameters, variables, and functions are as shown in Table 1. The superscript $d$ of $w_{h}^{d}$ represents selection caused by egg rejection. The term $(1- 1/n)$accounts for the fact that an egg is discarded upon parasitization. From Servedio and Lande (2003), it can be shown that

$$\begin{aligned} \Pr\left[ \mathrm{egg}i\mathrm{survives} \right|x_{i}=z_{h}, not parasitized]=1-\int_{-\infty}^{z_{h}} \delta\left( z_{h},z_{r} \right)\phi\left( z_{r},n-1 \right)dz_{r},\#\left( S2 \right) \end{aligned}$$

where

$$\begin{aligned} \phi\left( z_{r},n \right)=n\left[ F_{h}\left( z_{r} \right) \right]^{n-1}f_{h}\left( z_{r} \right),\#\left( S3a \right) \end{aligned}$$

and

$$\begin{aligned} \delta\left( z_{1},z_{2} \right)=\frac{1}{2}\mathrm{erf} \left[ \frac{z_{1}-z_{2}-b}{\sqrt{2}\nu} \right].\#\left( S3b \right) \end{aligned}$$

To calculate $\Pr\left[ \mathrm{egg}i\mathrm{survives} \right|x_{i}=z_{h}, parasitized]$, we need to consider whether the cowbird egg is the largest egg or not. When the cowbird egg is the largest, there are two cases: (1) $z_{c}>z_{h}>z_{r}$, where the host will discard the cowbird egg with probability $\delta\left( z_{c},z_{h} \right)$, and (2) $z_{c}>z_{r}>z_{h}$, where the host will discard the cowbird egg with probability $\delta\left( z_{c},z_{r} \right)$. The probability that the cowbird egg is the largest and egg $i$ will survive is included two terms that correspond to these situations respectively, such that (modified from Servedio and Lande 2003 eqn. 6)

$$\Pr\left[ \mathrm{egg}i survives, z_{c}\mathrm{largest} \right|x_{i}=z_{h}, parasitized]=$$

$$\int_{z_{h}}^{\infty} \left( \int_{-\infty}^{z_{h}} \phi\left( z_{r},n-2 \right)dz_{r} \right)f_{c}\left( z_{c} \right)\left[ \delta\left( z_{c},z_{h} \right)+\left( 1-\delta\left( z_{c},z_{h} \right) \right)s \right]dz_{c}$$

$$\begin{aligned} +\int_{z_{h}}^{\infty} \left( \int_{z_{r}}^{\infty} f_{c}\left( z_{c} \right)\left[ \delta\left( z_{c},z_{r} \right)+\left( 1-\delta\left( z_{c},z_{r} \right) \right)s \right]dz_{c} \right)\phi\left( z_{r},n-2 \right)dz_{r}.\#\left( S4 \right) \end{aligned}$$

This equation combines cases where the cowbird egg is discarded (with probability $\delta$) with the probability that the cowbird egg is not discarded but the host egg survives competition with the cowbird chick, with probability *s*(1- $\delta$). When the cowbird egg is not the largest egg, there are two situations; either the focal egg $i$ can be the largest egg, or one of the other host eggs can be the largest egg.

When the focal egg $i$ is the largest, there are two further cases: (1) $z_{h}>z_{c}>z_{r}$, where the host will discard the focal egg $i$ with probability $\delta\left( z_{h},z_{c} \right)$, and (2) $z_{h}>z_{r}>z_{c}$, where the host will discard the focal egg with probability $\delta\left( z_{h},z_{r} \right)$. The survival probability of egg $i$ in this case is

$$\Pr\left[ \mathrm{egg}i survives, z_{h}\mathrm{largest} \right|x_{i}=z_{h}, parasitized]=$$

$$\begin{aligned} s\int_{-\infty}^{z_{h}} \left( \int_{-\infty}^{z_{c}} \phi\left( z_{r},n-2 \right)dz_{r} \right)f_{c}\left( z_{c} \right)\left( 1-\delta\left( z_{h},z_{c} \right) \right)dz_{c}+ \\ s\int_{-\infty}^{z_{h}} \left( \int_{-\infty}^{z_{r}} f_{c}\left( z_{c} \right)dz_{c} \right)\phi\left( z_{r},n-2 \right)\left( 1-\delta\left( z_{h},z_{r} \right) \right)dz_{r}.\#\left( S5 \right) \end{aligned}$$

Note that equation (S5) only contains cases where the focal egg survives, which occurs with probability $s(1-\delta)$.

When one of the other host eggs is the largest (i.e., $z_{r}$ is the largest), there are two final cases: (3) $z_{r}>z_{c}>z_{h}$, where the host will discard another of its eggs (besides the focal one) with probability $\delta\left( z_{r},z_{c} \right)$, and (4) $z_{r}>z_{h}>z_{c}$, where the host will discard another of its eggs with probability $\delta\left( z_{r},z_{h} \right)$. However, in both cases, the cowbird egg and the focal egg $i$ will not be discarded, so the survival probability of egg $i$ conditioned on this situation is $s$. Therefore, we obtain

$$\Pr\left[ \mathrm{egg}i survives, z_{r}\mathrm{largest} \right|x_{i}=z_{h}, parasitized]=$$

$$\begin{aligned} s\left( \int_{z_{h}}^{\infty} \left( \int_{z_{c}}^{\infty} \phi\left( z_{r},n-2 \right)dz_{r} \right)f_{c}\left( z_{c} \right)dz_{c}+\int_{z_{h}}^{\infty} \left( \int_{-\infty}^{z_{h}} f_{c}\left( z_{c} \right)dz_{c} \right)\phi\left( z_{r},n-2 \right)dz_{r} \right).\#\left( S6 \right) \end{aligned}$$

By substituting equations (S2) though (S6) into equation (S1), we can obtain the survival probability $w_{h}^{d}(z_{h}|\overline{z}_{w},b)$ as a function of the focal egg size $z_{h}$, conditional on the mean host egg size $\overline{z}_{w}$ and the discrimination threshold $b$.

**Shift of the discrimination threshold through learning**

We assume that host females lower their threshold of discrimination $b$ once they discard an egg. We further assume the magnitude of the shift of the discrimination threshold, $D$, has a linear dependence on the size difference between the size of the largest and the second largest eggs, as

$$\begin{aligned} D\left( z_{1},z_{2} \right)=-\epsilon\left( z_{1}-z_{2} \right),\left( z_{1}>z_{2} \right),\#\left( S7 \right) \end{aligned}$$

Given a female with-nest mean $\overline{z}_{w}$ and discrimination threshold $b$, as noted above, a host female will discard an egg under two conditions, depending on whether the nest is parasitized or not. When the female’s nest is not parasitized, the female will discard an egg due to differences in the host egg size. Since the size of the different host eggs are independently distributed, the joint distribution of the largest host egg size $z_{1}$ and the second largest host egg size $z_{2}$ is

$$\begin{aligned} u\left( z_{1},z_{2} \right)=n\left( n-1 \right)f_{h}\left( z_{1} \right)f_{h}\left( z_{2} \right)\left[ F_{h}\left( z_{2} \right) \right]^{n-2},\left( z_{1}\geq z_{2} \right).\#\left( S8 \right) \end{aligned}$$

Therefore, the probability that an egg is discarded in the situation of no parasitism is

$$\begin{aligned} P_{NP}\left( \overline{z}_{w},b \right)=\Pr\left[ not parasitized \right]\times\Pr\left[ \mathrm{discard} | \overline{z}_{w},b, not parasitized \right]= \\ \left( 1-\frac{Cn_{c}}{H} \right)\int_{-\infty}^{\infty} \left( \int_{z_{2}}^{\infty} u\left( z_{1},z_{2} \right)\delta\left( z_{1},z_{2} \right)dz_{1} \right)dz_{2}.\#\left( S9 \right) \end{aligned}$$

The average shift of the discrimination threshold in the whole population contributed to by this situation is

$$\begin{aligned} D_{NP}\left( \overline{z}_{w},b \right)=\left( 1-\frac{Cn_{c}}{H} \right)\int_{-\infty}^{\infty} \left( \int_{z_{2}}^{\infty} D\left( z_{1},z_{2} \right)u\left( z_{1},z_{2} \right)\delta\left( z_{1},z_{2} \right)dz_{1} \right)dz_{2}.\#\left( S10 \right) \end{aligned}$$

When the female’s nest is parasitized, there are only $n-1$ host eggs left, and the distribution of the size of the largest host egg $z_{l}$ is $\phi(z_{l},n-1)$. There are then two cases: (1) $z_{l}>z_{c}$ and (2) $z_{c}>z_{l}$. The contribution of this situation to the probability of that an egg will be discarded is

$$P_{P}\left( \overline{z}_{w},b \right)=\Pr\left[ \mathrm{parasitized} \right]\times\Pr\left[ \mathrm{discard} | \overline{z}_{w},b, parasitized \right]=$$

$$\begin{aligned} \frac{Cn_{c}}{H}\left( \int_{-\infty}^{\infty} \left( \int_{z_{c}}^{\infty} \delta\left( z_{l},z_{c} \right)\phi\left( z_{l},n-1 \right)dz_{l} \right)f_{c}\left( z_{c} \right)dz_{c}+\int_{-\infty}^{\infty} \left( \int_{z_{l}}^{\infty} \delta\left( z_{c},z_{l} \right)f_{c}\left( z_{c} \right)dz_{c} \right)\phi\left( z_{l},n-1 \right)dz_{l} \right).\#\left( S11 \right) \end{aligned}$$

The average shift of the discrimination threshold contributed to by this situation is

$$\begin{aligned} D_{P}\left( \overline{z}_{w},b \right)= \\ \frac{Cn_{c}}{H}\int_{-\infty}^{\infty} \left( \int_{z_{c}}^{\infty} D\left( z_{l},z_{c} \right)\delta\left( z_{l},z_{c} \right)\phi\left( z_{l},n-1 \right)dz_{l} \right)f_{c}dz_{c}+ \\ \frac{Cn_{c}}{H}\int_{-\infty}^{\infty} \left( \int_{z_{l}}^{\infty} D\left( z_{c},z_{l} \right)\delta\left( z_{c},z_{l} \right)f_{c}dz_{c} \right)\phi\left( z_{l},n-1 \right)dz_{l}.\#\left( S12 \right) \end{aligned}$$

The discrimination threshold after learning occurs is $b^{'}=b+D_{NP}(\overline{z}_{w},b)+D_{P}(\overline{z}_{w},b)$. We denote the joint distribution of the within-nest mean $\overline{z}_{w}$ and discrimination threshold $b$ before learning as $f_{wb}(\overline{z}_{w},b)$.

**Selection differential of the host egg size**

We assume there is physiological stabilizing selection on egg size as

$$\begin{aligned} w_{h}^{p}\left( z_{h} \right)=\exp\left[ -\frac{\left( z_{h}-\theta_{h} \right)^{2}}{2{\omega_{h}}^{2}} \right].\#\left( S13 \right) \end{aligned}$$

The average fitness of a host egg conditional on $\overline{z}_{w}$ and $b$ is

$$\begin{aligned} w_{h}\left( z_{h}|\overline{z}_{w},b \right)=w_{h}^{d}\left( z_{h}|\overline{z}_{w},b \right)w_{h}^{p}\left( z_{h} \right).\#\left( S14 \right) \end{aligned}$$

Therefore, in the first and second clutch, the average fitness of a host egg of size $z_{h}$ are

$$\begin{aligned} w_{h}^{\left( 1 \right)}=\int_{-\infty}^{\infty} \int_{-\infty}^{\infty} \int_{-\infty}^{\infty} f_{h}\left( z_{h} | \overline{z}_{w} \right)w_{h}\left( z_{h}|\overline{z}_{w},b \right)f_{wb}\left( \overline{z}_{w},b \right)dz_{h}d\overline{z}_{w}db,\#\left( S15a \right) \end{aligned}$$

$$\begin{aligned} w_{h}^{\left( 2 \right)}=\int_{-\infty}^{\infty} \int_{-\infty}^{\infty} \int_{-\infty}^{\infty} f_{h}\left( z_{h} | \overline{z}_{w} \right)w_{h}\left( z_{h}|\overline{z}_{w},b^{'}\left( \overline{z}_{w},b \right) \right)f_{wb}\left( \overline{z}_{w},b \right)dz_{h}d\overline{z}_{w}db.\#\left( S15b \right) \end{aligned}$$

The average host egg size after selection from the first and second clutch, $\overline{z}_{h}^{(1)}, \overline{z}_{h}^{(2)}$, can be calculated by multiplying $z_{h}$ into the integrand of equation (S15). The overall selection differential is

$$\begin{aligned} S_{h}=\frac{\overline{z}_{h}^{(1)}+\overline{z}_{h}^{(2)}}{w_{h}^{\left( 1 \right)}+w_{h}^{(2)}}-\overline{z}_{h}.\#\left( S16 \right) \end{aligned}$$

**Selection differential of the discrimination threshold**

Since the discrimination threshold due to learning from the first clutch cannot be transmitted to the offspring generation, it requires us to track the original discrimination threshold of the female. In terms of the first clutch, females with a discrimination threshold $b$ will have fitness

$$\begin{aligned} w_{b}^{\left( 1 \right)}\left( b \right)=\int_{-\infty}^{\infty} \int_{-\infty}^{\infty} w_{h}\left( z_{h} | \overline{z}_{w},b \right)f_{wb}\left( \overline{z}_{w},b \right)f_{h}\left( z_{h}|\overline{z}_{w} \right)dz_{h}d\overline{z}_{w}.\#\left( S17 \right) \end{aligned}$$

To calculate the fitness of females at the second clutch, we denote the frequency of females whose within-nest mean egg size is $\overline{z}_{w}$ and the discrimination threshold value shifts from $b$ to $b^{'}$ by $P\left( \overline{z}_{w},b,b^{'} \right)$. Clearly, if $b^{'}=b$, there is no learning so that no egg is discarded in the first clutch. This situation occurs with a probability $1-P_{NP}-P_{P}$.

For the situation when an egg is discarded (so that $b^{'}<b$), there are two cases depending on whether parasitism occurs. When there is no parasitism, which occurs with probability $1-\frac{Cn_{c}}{H}$, we denote the size of the largest and the second largest host eggs by $z_{1}$ and $z_{2}$. Given fixed values of $\overline{z}_{w}$, $b$, and $z_{2}$, the probability that the discrimination threshold after learning $b'$ is no larger than a value $X$ is

$$\begin{aligned} P_{NP}\left( \overline{z}_{w},b,b^{'}\leq X,z_{2} \right)=\left( 1-\frac{Cn_{c}}{H} \right)\left( 1-\int_{z_{2}}^{z_{2}+\left( b-X \right)/\epsilon} \delta\left( z_{1},z_{2} \right)u\left( z_{1},z_{2} \right)dz_{1} \right),\#\left( S18 \right) \end{aligned}$$

where the upper limit of the integration, $z_{2}+(b-X)/\epsilon$, is obtained by solving $z_{1}$ from the discrimination threshold shift equation $b^{'}=b+D\left( z_{1},z_{2} \right)=X$. Note that equation (S18) is the cumulative distribution function (CDF) for $b^{'}$. By taking the first derivative with respect to $X$, we obtain the probability density function (PDF) as

$$\begin{aligned} P_{NP}\left( \overline{z}_{w},b,b^{'},z_{2} \right)=\frac{\partial P_{NP}\left( \overline{z}_{w},b,b^{'}\leq X,z_{2} \right)}{\partial X}\left. \right|_{X=b^{'}} \\ =\left( 1-\frac{Cn_{c}}{H} \right)\frac{1}{\epsilon}\delta\left( z_{2}+\frac{b-b^{'}}{\epsilon},z_{2} \right)u\left( z_{2}+\frac{b-b^{'}}{\epsilon},z_{2} \right).\#\left( S19 \right) \end{aligned}$$

When there is parasitism, there are two subcases, depending on whether the largest host egg is larger or smaller than the cowbird egg. Denote the size of the largest host egg by $z_{l}$ and the cowbird egg by $z_{c}$. When $z_{l}\geq z_{c}$, the PDF is

$$\begin{aligned} P_{P}^{\left( 1 \right)}\left( \overline{z}_{w},b,b^{'},z_{c} \right)=\frac{Cn_{c}}{H}f_{c}\left( z_{c} \right)\frac{1}{\epsilon}\delta\left( z_{c}+\frac{b-b^{'}}{\epsilon},z_{c} \right)\phi\left( z_{c}+\frac{b-b^{'}}{\epsilon},n-1 \right).\#\left( S20 \right) \end{aligned}$$

When $z_{c}>z_{l}$, the PDF is

$$\begin{aligned} P_{P}^{(2)}\left( \overline{z}_{w},b,b^{'},z_{l} \right)=\frac{Cn_{c}}{H}\phi\left( z_{l},n-1 \right)\frac{1}{\epsilon}\delta\left( z_{l}+\frac{b-b^{'}}{\epsilon},z_{l} \right)f_{c}\left( z_{l}+\frac{b-b^{'}}{\epsilon} \right).\#\left( S21 \right) \end{aligned}$$

Therefore, by summing up the above two cases, the frequency of females with within-nest mean $\overline{z}_{w}$ and a shift of the discrimination threshold from $b$ to $b'$ is

$$\begin{aligned} P\left( \overline{z}_{w},b,b^{'} \right)=\left( \int_{-\infty}^{\infty} P_{P}^{\left( 1 \right)}\left( \overline{z}_{w},b,b^{'},z_{c} \right)dz_{c}+\int_{-\infty}^{\infty} P_{P}^{\left( 2 \right)}\left( \overline{z}_{w},b,b^{'},z_{l} \right)dz_{l} \right)+ \\ \int_{-\infty}^{\infty} P_{NP}\left( \overline{z}_{w},b,b^{'},z_{2} \right)dz_{2}, \left( b^{'}<b \right).\#\left( S22 \right) \end{aligned}$$

As a summary, during the second clutch, the joint distribution of $\overline{z}_{w},b$ and $b'$ is:

$$\begin{aligned} g\left( \overline{z}_{w},b,b^{'} \right)=\left\{ \begin{aligned} 1-P_{NP}\left( \overline{z}_{w},b \right)-P_{P}\left( \overline{z}_{w},b \right), \left( b^{'}=b \right) \\ P\left( \overline{z}_{w},b,b^{'} \right), \left( b^{'}<b \right) \end{aligned} \right..\#\left( S23 \right) \end{aligned}$$

Therefore, during the second clutch, the average fitness of females with an original discrimination threshold $b$ is

$$\begin{aligned} w_{b}^{\left( 2 \right)}\left( b \right)=f_{b}^{-1}\left( b \right)\int_{-\infty}^{\infty} \int_{-\infty}^{\infty} \left( \int_{-\infty}^{b} w_{h}\left( z_{h} | \overline{z}_{w},b^{'} \right)f_{wb}\left( \overline{z}_{w},b \right)g\left( \overline{z}_{w},b,b^{'} \right)db^{'} \right)f_{h}\left( z_{h}|\overline{z}_{w} \right)dz_{h}d\overline{z}_{w}.\#\left( S24 \right) \end{aligned}$$

The overall selection differential of the discrimination threshold is

$\begin{aligned} S_{b}=\frac{\int_{-\infty}^{\infty} b\left( w_{b}^{\left( 1 \right)}\left( b \right)+w_{b}^{\left( 2 \right)}\left( b \right) \right)f_{b}\left( b \right)db}{w_{h}^{\left( 1 \right)}+w_{h}^{\left( 2 \right)}}-\overline{b}.\#\left( S25 \right) \end{aligned}$

**Selection differential of the cowbird egg size**

We also assume the cowbird egg size is subject to physiological stabilizing selection $w_{c}^{p}(z_{c})$, with the optimum $\theta_{c}$ and strength $1/\omega_{c}^{2}$. Following Servedio and Lande (2003), the viability due to discrimination of the host is

$$\begin{aligned} w_{c}^{d}\left( z_{c} \right)=\int_{-\infty}^{\infty} \int_{-\infty}^{\infty} \Pr\left( cowbird egg j survives|x_{j}=z_{c} \right)f_{wb}\left( \overline{z}_{w},b \right)d\overline{z}_{w}db,\#\left( S26 \right) \end{aligned}$$

where

$$\begin{aligned} \Pr\left( cowbird egg j survives|x_{j}=z_{c} \right)=\int_{-\infty}^{z_{c}} \phi\left( z_{r},n-1 \right)\left[ 1-\delta\left( z_{c},z_{r} \right) \right]dz_{r}+1-\left[ F_{h}\left( z_{c} \right) \right]^{n-1}.\#\left( S27 \right) \end{aligned}$$

The overall fitness of a cowbird egg with size $z_{c}$ is $w_{c}^{(1)}\left( z_{c} \right)=w_{c}^{d}\left( z_{c} \right)w_{c}^{p}\left( z_{c} \right)$. For the second clutch, we just need to replace $f_{wb}$ with $f_{wb}'$ in the expression of $w_{c}^{d}$, and the fitness in the second clutch is denoted as $w_{c}^{(2)}$. The selection differential on the cowbird egg size is

$$\begin{aligned} S_{c}=\frac{\int_{-\infty}^{\infty} z_{c}f_{c}\left( z_{c} \right)\left[ w_{c}^{\left( 1 \right)}\left( z_{c} \right)+w_{c}^{\left( 2 \right)}\left( z_{c} \right) \right]dz_{c}}{\overline{w}_{c}}-\overline{z}_{c},\#\left( S28 \right) \end{aligned}$$

where the overall fitness is

$$\begin{aligned} \overline{w}_{c}=\int_{-\infty}^{\infty} f_{c}\left( z_{c} \right)\left[ w_{c}^{1}\left( z_{c} \right)+w_{c}^{2}\left( z_{c} \right) \right]dz_{c}.\#\left( S29 \right) \end{aligned}$$
